# Microstructure and Mechanical Properties of Nano-Carbon Reinforced Titanium Matrix/Hydroxyapatite Biocomposites Prepared by Spark Plasma Sintering

**DOI:** 10.3390/nano8090729

**Published:** 2018-09-15

**Authors:** Feng Li, Xiaosong Jiang, Zhenyi Shao, Degui Zhu, Zhiping Luo

**Affiliations:** 1 School of Materials Science and Engineering, Southwest Jiaotong University, Chengdu 610031, China; lifengswjtu@yeah.net (F.L.); dgzhu@home.swjtu.edu.cn (D.Z.); 2 Department of Chemistry and Physics, Fayetteville State University, Fayetteville, NC 28301, USA; zluo@uncfsu.edu

**Keywords:** titanium alloy, hydroxyapatite, nano-carbon, microstructure, biocompatibility

## Abstract

Nano-carbon reinforced titanium matrix/hydroxyapatite (HA) biocomposites were successfully prepared by spark plasma sintering (SPS). The microstructure, mechanical properties, biocompatibility, and the relationship between microstructure and properties of biocomposites were systematically investigated. Results showed there are some new phases in sintered composites, such as β-Ti, TiO_3_, ZrO_2_, etc. Moreover, a small amount of Ti_17_P_10_, CaTiO_3_, Ca_3_(PO_4_)_2_ were also detected. The reaction that may occur during the preparation process is suppressed to some extent, which is because that the addition of second phases can prevent the direct contact of titanium with HA and reduce the contact areas. Transmission electron microscope (TEM) analysis proved the existence of elemental diffusion and chemical reactions in sintered composites. Compared with results of composites prepared by hot-pressed sintering before, mechanical properties (microhardness, compressive strength, and shear strength) of 0.5-GNFs composites prepared by SPS were increased by about 2.8, 4.8, and 4.1 times, respectively. The better mechanical properties of 0.5-GNFs composite in nano-carbon reinforced composites are mainly due to the lower degree of agglomeration of tubular carbon nanotubes (CNTs) compared to lamellar graphene nanoflakes (GNFs). Moreover, the strengthening and toughening mechanisms of nano-carbon reinforced titanium alloy/HA biocomposite prepared by spark plasma sintering (SPS) mainly included second phase strengthening, grain refinement strengthening, solution strengthening, graphene extraction, carbon nanotubes bridging, crack tail stripping, etc. In addition, in vitro bioactivity test revealed that the addition of nano-carbon was beneficial to promote the adhesion and proliferation of cells on the surface of titanium alloy/HA composite, because nano-carbon can enhance the formation of mineralized necks in the composites after transplantation, stimulate biomineralization and promote bone regeneration.

## 1. Introduction

The specific gravity of titanium alloy, which has excellent mechanical properties, is close to that of human bone, but it is mechanically fitted with bone tissue in the living body. Therefore it is easy to have poor bonding with bones and may even fall off after being implanted into the body [1,2]. Hydroxyapatite (HA) is the main inorganic component of bone tissue, which can form strong active connections with bone tissue through chemical bonding in a biological environment [3,4,5]. However, the poor mechanical properties of HA greatly affect clinical application [6,7]. Therefore, the composites composed of titanium alloys and HA are prepared by various processes for practical application. However, it has its intrinsic limitations and disadvantages in the prepared coating composites, such as high porosity, uneven thickness distribution, low crystallinity, weak bonding strength, etc. [8,9]. Singh et al. found that the bond strength of HA-Al_2_O_3_, HA-ZrO_2_ coating with Ti-6Al-4V substrate reduce after heat treatment [9]. This was also related to the diffusion of oxygen in the titanium alloy matrix, except the difference in thermal expansion coefficient between titanium alloy matrix and HA. A hard and brittle oxygen-rich layer was formed when oxygen diffused into the surface of the substrate. The existence of oxygen-rich layer reduces interfacial bonding strength of matrix and coating. Moreover, Miranda et al. successfully prepared Ti-6Al-4V/HA composites by powder metallurgy, and the results showed that HA can be evenly distributed in the titanium alloy matrix and tightly bonded with the matrix even after the shearing experiment, which avoids the problem of weak metal-ceramic interface bonding in the coating composites [5]. Nevertheless, there are the following problems in uncoated composites: (1) stress shielding phenomenon [5,7,10]; (2) Violent reactions between HA and Ti during high temperature sintering [11,12,13]; (3) The large difference in thermal expansion coefficient between HA and titanium alloy [14,15].

In fact, a new type of biological material that satisfies its comprehensive performance can be obtained by the adjustment of preparation method and components [16,17,18,19]. Research showed that carbon nanotubes (CNTs) and graphene nanoflakes (GNFs) not only have high strength, specific surface area, corrosion resistance and other properties, but also appear good biocompatibility and degradability [20,21,22]. After added in composites, CNTs and GNFs are expected to improve biocompatibility of composites while maintaining their mechanical properties [20,21,22]. It was revealed that both CNTs-HA and graphene sheet-HA composites are ideal materials for bone tissue engineering scaffolds and beneficial to the proliferation and differentiation of human fetal osteoblastic cell line (hFOB 1.19) [23]. Yang et al. found that compressive strength of graphene-Ti composites improve with the addition of an appropriate amount of graphene, which is also partly due to the enhancement effect of graphene itself [24]. However, the agglomeration of graphene occurred around some pores present on the surface of the composite [24]. On the other hand, an appropriate amount of CNTs and GNFs have synergistic enhancement effects due to their unique structure [25,26]. Theoretically, one-dimensional CNTs and two-dimensional GNPs can form three-dimensional mixed space, which can promote dispersion of nano-carbon and increase contact areas with matrix [25,26]. Researchers revealed that carbon nanotubes, which act as scaffolds in three-dimensional graphene oxide-carbon nanotube polyaniline hybrids, are surrounded by graphene and form the three-dimensional structure [27]. However, the uniform distribution of nano-carbon in the composites after sintering is a challenge. Therefore, researchers often modify the surface of nano-carbon by physical dispersion and chemical dispersion to reduce its agglomeration [28,29,30].

In addition, studies showed that the addition of lanthanum can not only capture the oxygen of composites, but also promote grain refinement [31]. Lou et al. revealed that the lanthanum in the lanthanum-doped HA coating partially replaces calcium ions in HA, which changes crystal structure of HA and increases crystallinity of HA to some extent [32]. Spark plasma sintering (SPS) is an advanced sintering method based on pressure-assisted sintering of inter-particle spark transient discharge sintering. During sintering process, the discharge plasma generated instantaneously when the electrode is supplied with a direct current pulse current, which make particles inside the sintered body uniformly generate Joule heat and surface activation. Therefore, the plasma effect, Joule heat, electric field diffusion effect, etc. in the process of spark plasma sintering can promote the diffusion of atoms between composites and finish sintering within a few minutes [16]. In this paper, nano-carbon reinforced Titanium matrix/HA composites were prepared by spark plasma sintering technology for obtaining a more homogeneous, dense, and high quality sintered body. An appropriate amount of lanthanum also been added in the composites. On this basis, the microstructure and mechanical properties of composites was analyzed by various methods and machines. Based on the characterization of microstructure and mechanical properties, the strengthening and toughening mechanisms of composites was analyzed. Besides, in vitro cell compatibility of composites was also tested by staining analysis and scanning electron microscope (SEM), and the relationship between microstructure and biological properties was also analyzed.

## 2. Materials and Methods

**Figure 1:**
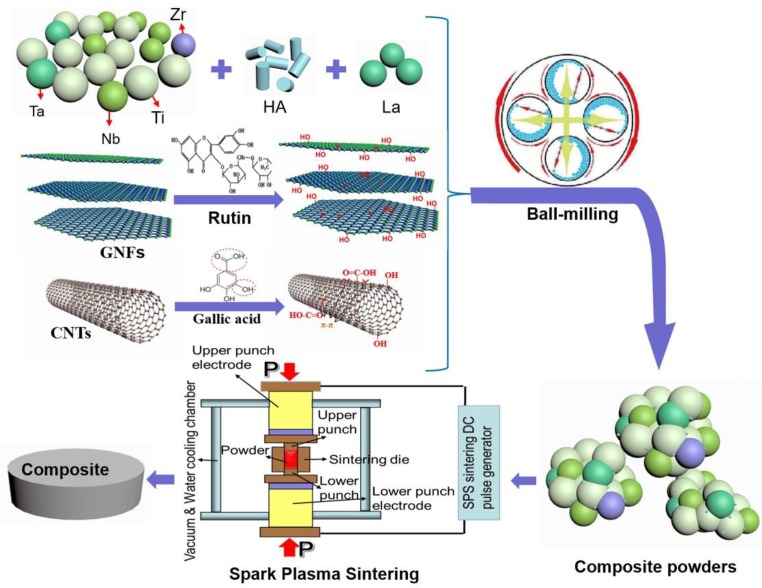
The schematic diagram of the experiment.

The ingredients list of this experiment is shown in Table 1. Metal powders (titanium, tantalum, niobium and zirconium, purity greater than 99.5%, 300 mesh, Beijing Xing Rong Yuan Technology Co., Ltd., Beijing, China), HA powders (average diameter 40 nm, needle-like, Xi’an Bella Biotechnology Co., Ltd., Xi’an, China), La (Beijing Xing Rong Yuan Technology Co., Ltd., Beijing, China), CNTs (Chengdu Organic Chemicals Company Ltd. of Academy of Sciences, Chengdu, China) and GNFs (Shanghai Chaohao New Material Technology Co., Ltd., Shanghai, China) were used in the experiment. Firstly, CNTs and GNFs were carried out on surface modification using aqueous solution of gallic acid and rutin, respectively [28,29]. Then after weighing various materials according to Table 1, mechanical ball milling (5 h), freeze drying and SPS were sequentially performed. Spark plasma sintering was carried out in the vacuum environment at 1000 °C with the heating rate of 100 °C/min. The composite was kept for a sintering time of 5 min and the pressure of 50 MPa, then the furnace was naturally cooled to room temperature. The schematic diagram of experiment is listed as Figure 1.

The sintered composites is tested after the process of wire cut of electric discharge, ground using emery paper, polishing. Phase compositions of powders and sintered samples were measured using X-ray diffraction (XRD, X-Pert PRO-MPD, PANalytical B.V., Almelo, The Netherlands). The microstructure and compositions of milled powders and sintered samples were analyzed by SEM (JSM-6610 LV, JEOL, Tokyo, Japan) and Electron Probe Microanalyzer (EPMA, JXA-8230, JEOL, Tokyo, Japan). Transmission electron microscopy (TEM, Tecnai F20ST, FEI, Hillsboro, OR, USA) was used to further analyze the microstructure of sintered composites. In terms of mechanical properties, the density, microhardness were measured by the Archimedes method and Vickers microhardness tester (Micro-586, Donghua, Shanghai, China) with the load of 500 g and holding time of 10 s. Compressive and shear strength of composites were tested using a microcomputer control electron universal testing machine (WDW-3100, Changchun Kexin Test Instrument Co., Ltd., Changchun, China) with a movement rate of 0.1 mm/min. The morphology and characteristics of fracture surface were analyzed by SEM equipped with energy dispersive X-ray spectroscopy (EDS). The bending strength were analyzed by a three point bending test (WDW-3100, Changchun Kexin Test Instrument Co., Ltd., Changchun, China) with the samples of 20 mm × 3 mm × 2.5 mm, load of 0.5 kN load with 0.5 mm min^−1^ cross-head speed and span of 12 mm. The strength value is obtained by measuring three samples of each component and taking the average value.

In vitro biocompatibility of nano-carbon reinforced titanium matrix/HA composites for MC3T3-E1 cells was evaluated by phalloidin staining and SEM [33,34,35]. Firstly, the composites processed into a circular of 10 mm diameter using wire cutting were immersed in 75% ethanol for 1 h after being ground, polished, ultrasonically cleaned (ethanol, acetone), and then immersed in α-MEM (α-minimum Eagle’s medium) to remove alcohol.MC3T3-E1 cells (institute of orthopaedics at Soochow University) that have been cultured were seeded in a 24-well plate (Corning) on the surface of the composite at a density of 5 × 10^4^/well. After the cells were cultured in an incubator for 24 h, the culture solution was removed. Then the cells were washed three times with phosphate buffer salin (PBS), fixed with 4% paraformaldehyde for 15 min, and dehydrated with alcohol gradient (30%, 50%, 70%, 70%, 85%, 90%, 100%). After the process of critical point drying and gold spraying, the cell adhesion and proliferation morphology were analyzed by SEM. On the other hand, each group of composites was immersed in 75% alcohol for 1 h, then the alcohol was removed by soaking in α-MEM. Next, each set of materials were placed into a 24-well plate, and MC3T3-E1 cells were seeded on the surface of materials in the 24-well plate (5 × 10^4^ cells per well). After adding α-MEM + 10%FBS (fetal bovine serum) + 1%P/S (Penicillin-Streptomycin), the cells were cultured in an incubator for 24 h. Finally, the cells were stained with Dapi and phalloidin (Invitrogen, Carlsbad, CA, USA) and observed by fluorescence microscopy (TH4-200, Olympus, Tokyo, Japan).

## 3. Results

### 3.1. Microstructure and Phase Composition of Nanocomposites Powder

**Figure 2:**
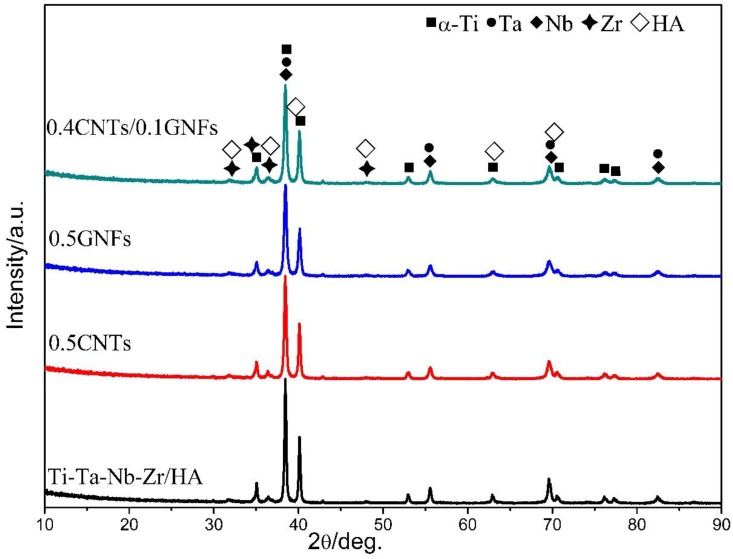
X-ray diffractograms of milled powders.

Figure 2 showed the XRD spectra of milled composites. Research revealed that the presence of Ti, Ta, Nb, Zr, and HA can still be detected in the mixed powders after ball milling, and no other new phases appeared. Therefore it can been found that typical chemical reactions do not occur during high energy mechanical ball milling, which is similar to the result of Bovand et al. [11]. However, some researchers have found that cold welding and pulverization process generated from the high-energy mechanical ball milling can also cause decomposition of HA to form CaHPO_4_ [12]. There was no significant difference in the XRD patterns obtained from different milled composite powders, because the added phases La, CNTs and GNFs were not detected in the XRD patterns due to their low content.

**Figure 3:**
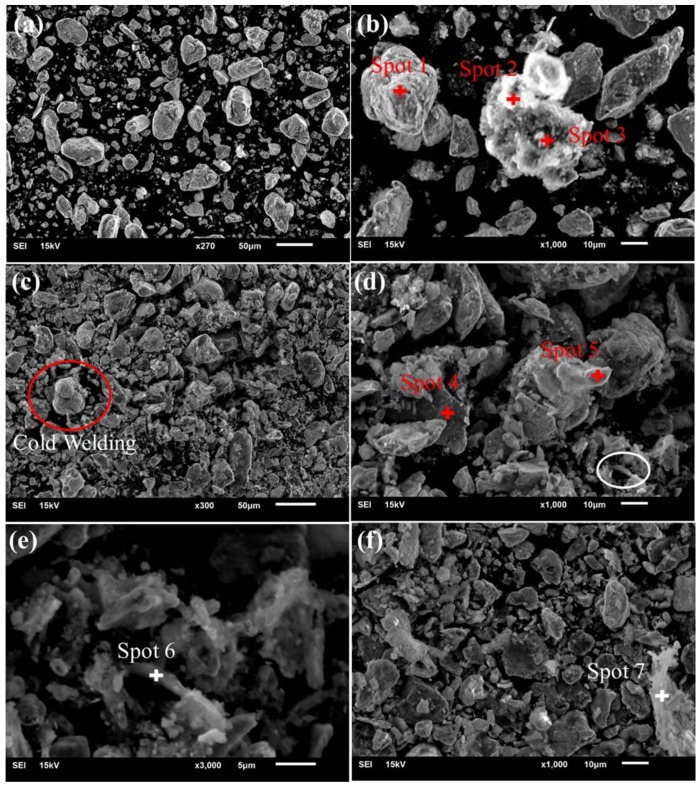
(**a**,**b**) Scanning electron microscope (SEM) micrographs of the 0.5-CNT snanocomposites in different regions; and (**c**–**e**) SEM micrographs of the 0.5-GNFs nanocomposites in different regions; (**f**) SEM micrographs of the 0.4 CNTs: 0.1 GNFs nanocomposites.

Figure 3 and Table 2 show the microscopic morphology and composition of composite powders after ball milling. It can be found from Figure 3 that the particle size of milled powders is about 5 μm–30 μm compared with the average particle size (about 45 μm) of original powders, which is caused by the continuous cold welding-grinding process during ball milling [36]. However, research also revealed that refined particles will reunite when the ball milling time is too long [12]. It was found from Table 2 and Figure 3 that the elements types at points 1, 2, and 3 in Figure 3b are similar and contain C, O, Ti, P, Ca, etc. The content of C element was the highest at point 2, and C here was the carbon nanotube. Therefore, it was revealed that carbon nanotubes were coated by titanium particles, and HA particles were partly distributed on the surface of matrix particles. Similarly, researchers have found that nano-HA is uniformly coated on the outside of alloy particles to form a spherical shell structure after ball milling, which is mainly due to the fracture and cold welding effect during high-energy ball milling [37]. The particles in the red circle in Figure 3c exhibited the characteristics of ball-milled cold welding, which was a normal phenomenon. The Ti, Nb, C, and Ta were the main elements at the spots 4 and 5 in Figure 3d. It can be seen from Figure 3d that the milled particles may form a sheet. Akmal et al. also found that TiNi/HA composites change from original spherical and flaky materials to the layered material after ball milling, which is mainly caused by the combination of grain refining and cold welding in the ball milling process [13]. Figure 3e is a magnified image of the white circle area in Figure 3d, and it was revealed from Table 2 that the layered material of spot 6 and 7 is GNFs. Moreover, HA particles and a small amount of metal element particles were distributed on the layered materials of spot 6 and 7 in Figure 3e,f.

### 3.2. Microstructure and Phase Analysis of the Sintered Nanocomposites

#### 3.2.1. XRD Results Analysis

**Figure 4:**
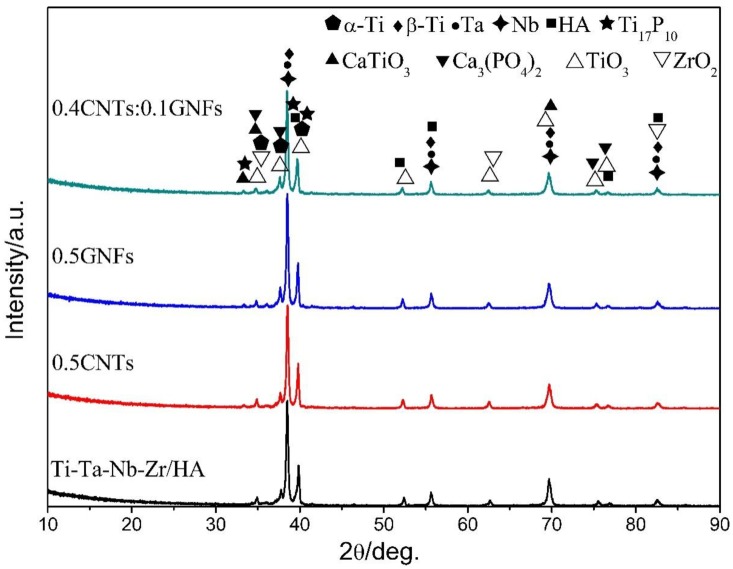
X-ray diffraction patterns (XRD) of nanocomposites sintered at 1000 °C.

It can be seen from Figure 4 that the sintered composites contain α-Ti, β-Ti, Ta, Nb, HA, Ti_17_P_10_, CaTiO_3_, Ca_3_(PO_4_)_2_, TiO_3_, ZrO_2_, which showed that chemical reactions occur in the composite during SPS sintering [15,16]. Moreover, β-Ti was formed by the phase transition of original α-Ti during the sintering process and then was remained due to the existence of a β-state stable phase such as Ta, Nb, etc. Because the existence of β-state stable phase can reduce the phase transition temperature of titanium, enlarge the β phase region, and increase the thermodynamic stability of β phase. The original α-Ti phase still existed in the sintered composites and it was seen that there is only a weak peak belonging to Ti_17_P_10_ and CaTiO_3_ at a position of about 33° on the XRD spectra of nano-carbon reinforced titanium matrix/HA composite. Therefore, it was considered that there is no significant difference in the XRD spectra of different sintered samples, and the existence of La, GNFs, and CNTs phases added in the sintered samples are not detected by XRD due to their low content. There were only high-intensity peaks of Ta and Nb detected in sintered composites, and no new phases formed by Ta or Nb were found in the XRD results, which indicated that no chemical reactions occur concerning Ta and Nb during the sintering process. In addition, the XRD did not detect the reactions between titanium and nano-carbon, which indicated that the vacuum environment effectively protects the purity of sintering process. Moreover, researchers also found that the pyrolysis of oxygen-containing functional groups occurs during the preparation of graphene-reinforced titanium-based composites by laser sintering [22]. But no carbides and oxides were found, and some graphene oxide remained after sintering [22].

#### 3.2.2. SEM Results Analysis

**Figure 5:**
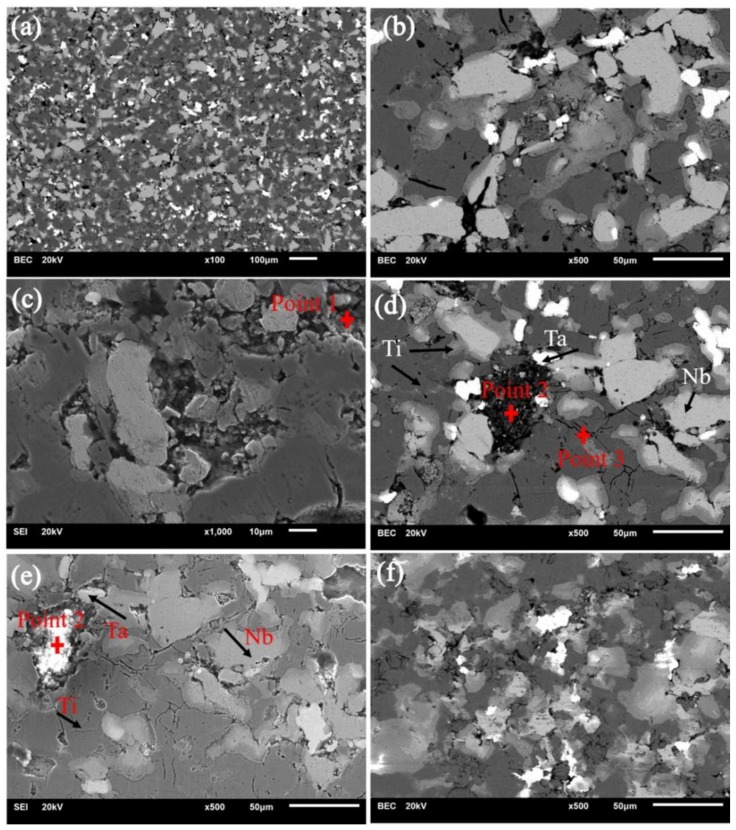
SEM micrographs of sintered nanocomposites in different regions. (**a**,**b**) Ti-Ta-Nb-Zr/HA; (**c**) 0.5 CNTs; (**d**,**e**) 0.5 GNFs; (**f**) 0.4 CNTs/0.1 GNFs.

Figure 5 showed secondary electron and backscattered electron images of sintered composite, and it can be seen from Figure 5a that various components are intertwined and approximately evenly distributed. The backscattered electron morphology of sintered composite consisted of four different contrast images, as shown in Figure 5d. The dark gray areas (84 Ti, 7.51 C, 7.91 O, 0.24 Nb, 0.20 La, 0.05 Ca, 0.04 P, 0.05 Ta, wt %) was the largest, and its main elements were titanium and carbon. The gray areas (52.90 Ti, 21.75 Nb, 8.41 Zr, 9.48 C, 7.29 O, 0.15 P, 0.02 Ca, wt %) also contained titanium, but the content of Nb and Zr was higher. The light gray areas (75.13 Nb, 17.81 C, 6.28 O, 0.43 P, 0.27 Ti, 0.07 Ca, wt %) mainly contained Nb and C, and the main element of bright white areas (85.54 Ta, 10.98 C, 3.34 O, 0.48 Zr, 0.36 Ti, 0.23 Nb, 0.07 Ca, wt %) was Ta. These areas constituted the main parts of the microstructure of sintered composites. The reason for the higher C content in these areas was that the distribution of nano-carbon in the composites is concentrated and agglomerated. The secondary electron images of the composite were composed mainly of three different color images, as shown in Figure 5e. There were also some shedding phases and fragmentation phases in the region of spots 1 and 2 whose chemical composition was shown in Table 3, which resulted from the processes of grinding and polishing during preparation. In addition, it was seen from Figure 5b,d that some micropores and cracks are mainly present in the distribution areas of titanium elements. Zhu et al. also revealed that alloy particles are bonded to each other by the formed sintered necks in Ti_40_Zr_10_Cu_36_Pd_14_-HA composites prepared SPS, and there is almost no obvious interface [38]. A small amount of pores were present on the surface of the composites, but the surface of the composite became uneven and partial segregation occurred when the HA content is higher than 3% [38]. This was because of the low thermal conductivity of HA, decomposition of HA during sintering, and partial crystallization of the glass phase alloy matrix [38]. 

#### 3.2.3. EPMA Results Analysis

**Figure 6:**
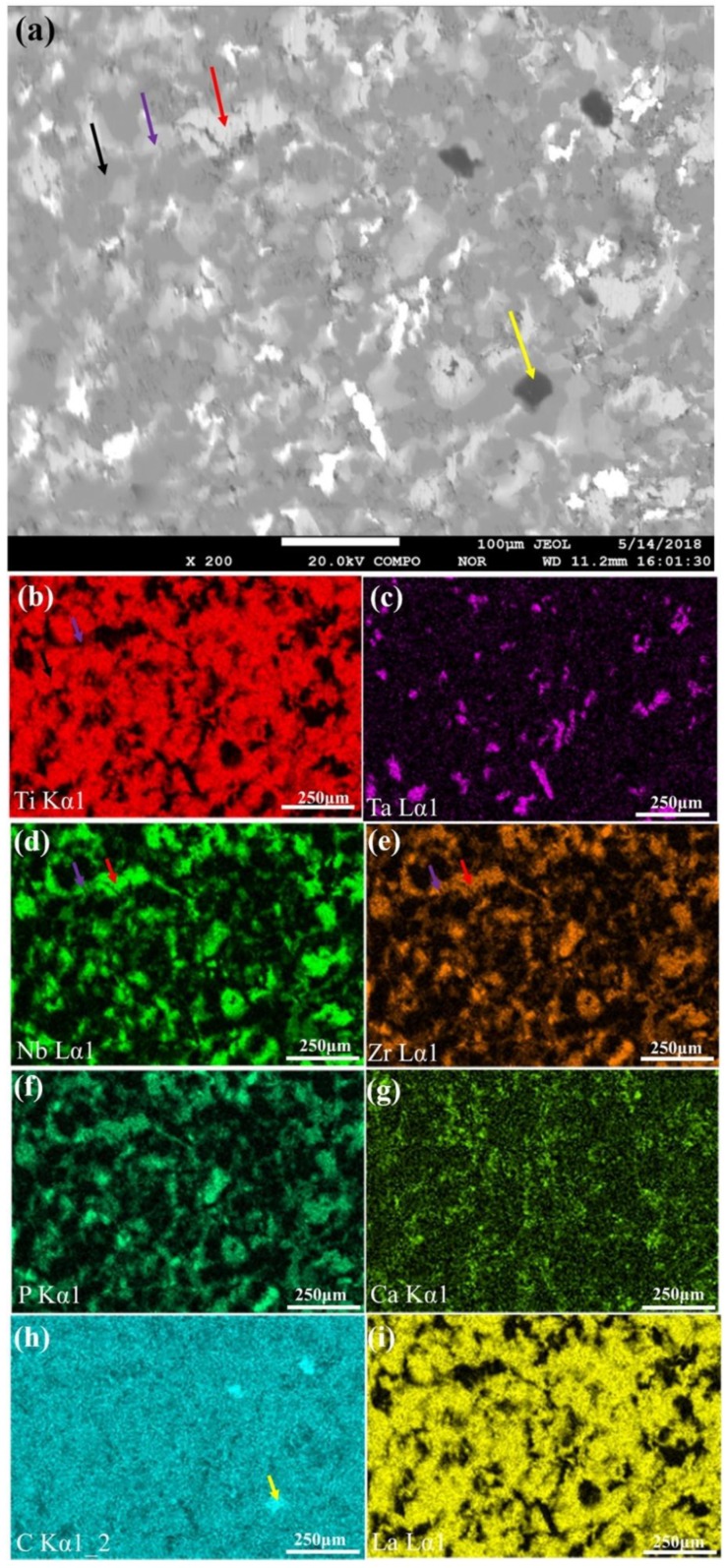
(**a**) SEM micrographs of the 0.5-GNFs nanocomposite sintered at 1000 °C. The corresponding mapping scanning results of EPMA: (**b**) Ti; (**c**) Ta; (**d**) Nb; (**e**) Zr; (**f**) P; (**g**) Ca; (**h**) C; and (**i**) La. According to the Siegbahn nomenclature, Kalpha 1 and Lalpha 1 refers to the name of the electronic transition when exciting X-rays. Kalpha refers to the electronic transition from the L-layer to the K-layer. Lalpha refers to the electronic transition from the M-layer to the L-layer.

It was seen from Figure 6 that compared with Figure 5d, there are four areas with different shade degree, which is because of the difference in atomic number. When atomic number is larger, the number of backscattered electrons produced is more and the corresponding graph is brighter. The composition of bright gray areas (marked by red arrows) were mainly Nb and Zr whose atomic numbers are similar. It can be seen that the areas of Nb and Zr are almost coincident, which showed that they may form solid solution. He et al. revealed that the angular shift of titanium peak in the XRD of (Ti-13Nb-13Zr)-10 HA composites is mainly due to the changes of crystal parameters caused by solid solution and sintering defects [13]. In addition, the formation of Ti (Nb) and Ti (Zr) supersaturated solid solution not only changed crystal parameters of titanium, but also improved performance due to solution strengthening [13]. The areas indicated by purple arrows mainly contained Nb, Zr and Ti, so the brightness was slightly brighter than the gray titanium area. The distribution of La element was similar to that of titanium and distributed evenly on the surface of composites. A small amount of black area enrichment zones of C element was distributed on the surface of composites. The distribution of Ca and P elements was similar, which indicated the presence of calcium-phosphorus compounds. The distribution of Ca elements was more sparse, compared with that of P elements, which was due to the abscission of calcium-containing phases in the process of the sample preparation. The distribution of P element was similar to that of Nb and Zr elements, indicating that there was the element diffusion between each other in the sintered composites [39,40]. Moreover, phosphorus ions diffused into the Nb and Zr matrix, which also affects the calcium/phosphorus ratio of the apatite, and researchers revealed that phosphorus ions can migrate rapidly into the Ti substrate due to its small radius and low activation energy [10].

#### 3.2.4. TEM Results Analysis

**Figure 7:**
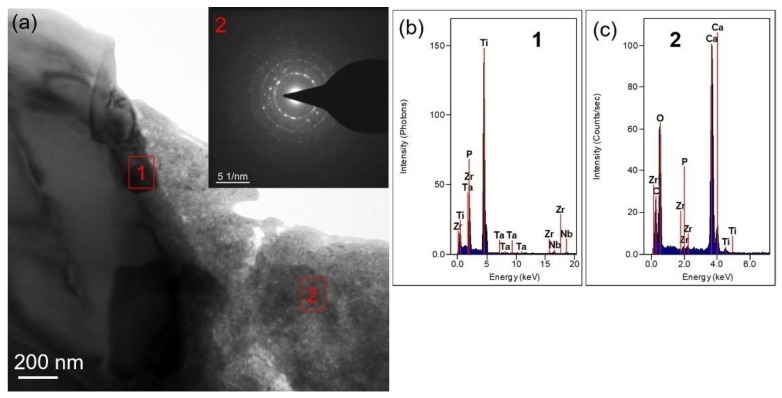
(**a**) Transmission electron microscope (TEM) image of the 0.5-GNFs nanocomposite prepared by SPS. The inset shows the selected area diffraction (SAD) pattern of spot 2; (**b**,**c**) the corresponding EDS results of spots 1 and 2.

**Figure 8:**
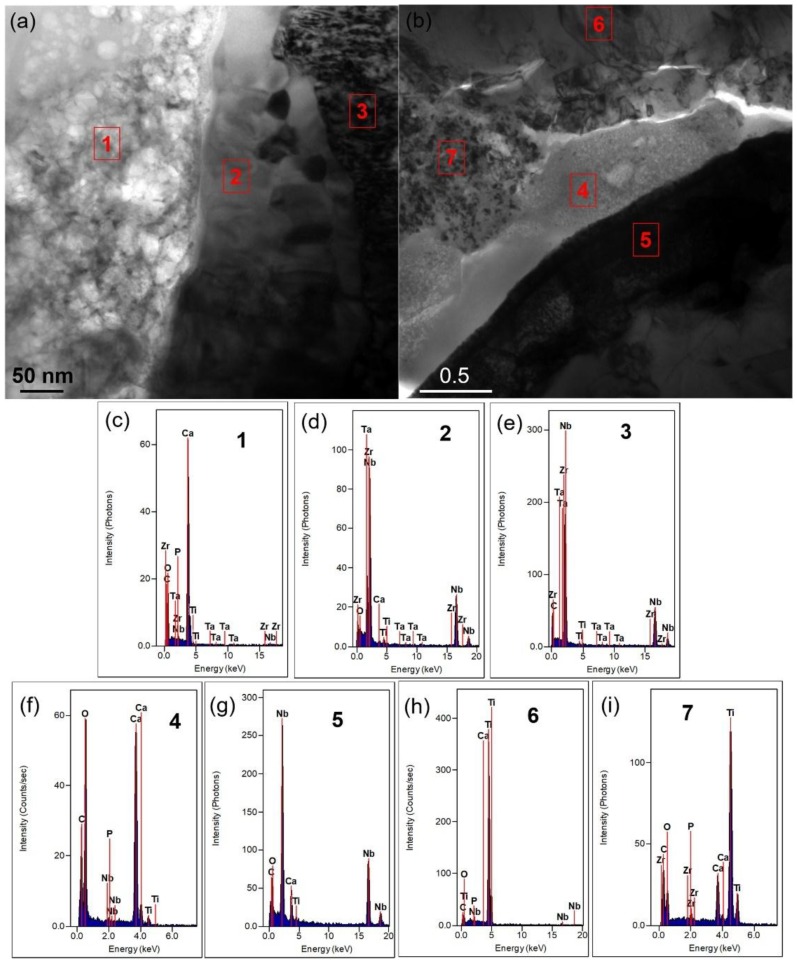
(**a**,**b**) TEM image of the 0.5-GNFs and 0.4 GNFs/0.1-CNTs nanocomposite in different areas, respectively. (**c**–**i**) the corresponding EDS spectrums of spots 1–7.

TEM was used to further analyze the microstructure and composition of the composite, and Figure 7a is the morphology and diffraction results of 0.5-GNFs composite. Combined with the EDS results of Figure 7b, it was known that there are matrix phases composed of Ti, P, Ta, Nb, Zr, etc. and calcium-containing reactants composed of Ca, P, O, C, Ti, Zr, etc. in the sintered composites. In terms of composition, the calcium-containing reactants may include HA, CaO, CaCO_3_, CaTiO_3_, Ca_3_(PO_4_)_2_, etc., and matrix phases contained Ti, Ta, Nb, Zr, Ti*_x_*P*_y_* and so on [12,17,41]. The composition of the matrix phases and reactants was similar to the results of XRD and SEM described above. The difference of the shade degree of colors in the matrix phases was mainly due to the mass thickness contrast. The discontinuity phenomenon in the diffraction ring (Figure 7a) of calcium-containing reactants was because of the small number of crystal grains and the coarse grain, and it was seen from the diffraction image and EDS results (Figure 7) that the calcium-containing reactants exhibits a mixture structure of amorphous and composite crystals [29]. Figure 8 further demonstrates the different morphologies and interface combinations present in the composite. It was seen that the composite also mainly contains calcium-containing reactants and matrix phases, which is similar to the results of Figure 7. But due to the difference of mass thickness contrast, different morphologies are formed in the composites. As shown in Figure 8a,b, the spots 1 and 4 contained calcium-containing reactants, and the areas of spots 2, 3, 5, 6, and 7 mainly were matrix phases. While the components of matrix phases were similar, but different morphologies there were mainly caused by different element contents that showed in Figure 8c–i. In addition, the complexity of elements contained in the matrix and calcium-containing reactants also reflected the existence of interdiffusion of elements during sintering. Researchers revealed that graphene, uniformly distributed in the titanium matrix, still exists in the titanium matrix/multilayer graphene nanofiller composites prepared by SPS, despite the reactions between nano-carbon and titanium that occur during high-temperature sintering [21]. This indicated that it is possible that only the nano-carbon at the ends or defects react with titanium due to the presence of the vacuum environment and surface modification of nano-carbon, and the amount of nano-carbon that has reacted with titanium is very small [20]. Moreover, it was seen from Figure 7 and Figure 8 that C element is almost present in both the matrix phases and calcium-containing reactants, which indicates that the nano-carbon may be coated by matrix phases and calcium-containing reactants.

### 3.3. Mechanical Properties of the Sintered Nanocomposites

**Figure 9:**
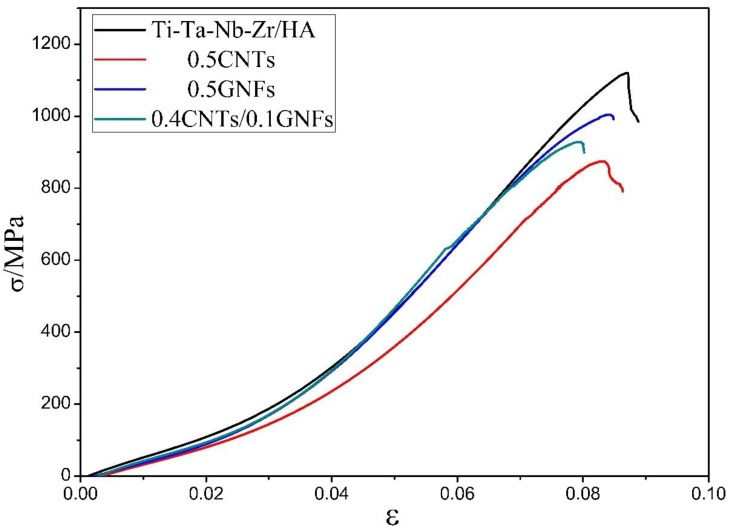
Compressive stress-strain curves of nanocomposites sintered at 1000 °C.

**Figure 10:**
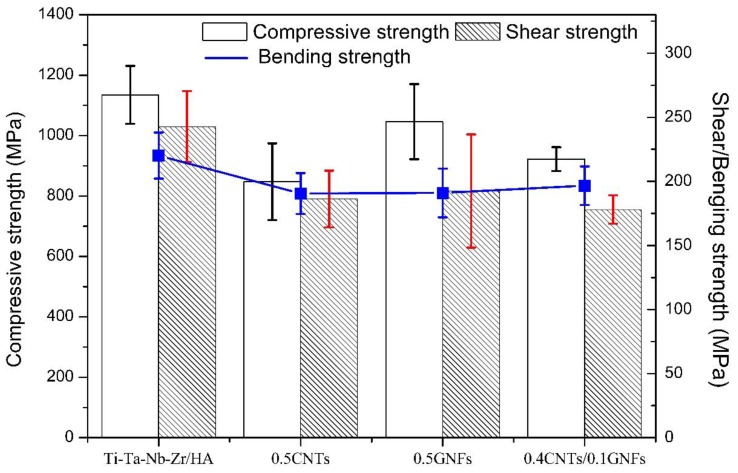
Compressive, shear and bending strength of the nanocomposites.

Figure 9 shows the compressive stress-strain curve of sintered composites, and it was seen that there is no obvious yielding phenomenon in the curve. Table 4 and Figure 10 compare the various mechanical properties of composites after sintering. It was found that the density of sintered composites, reaching more than 98%, has been effectively improved, and the difference between different samples is not large. The high density is beneficial to the increase of mechanical properties of composites to a certain extent. In fact, compared with the performance of composites prepared by vacuum hot press sintering [29]. The 0.5-GNFs reinforced titanium alloy/HA composite prepared by SPS had a density of 98% (about 80%, hot pressing sintering [29]). Moreover, the microhardness, compressive strength, and shear strength of 0.5-GNFs composite prepared by SPS were improved by about 2.8, 4.8, and 4.1 times, respectively, which is because of the advanced nature of SPS sintering process and the change of sample composition [29]. The flexural strength of sintered composites prepared by SPS had also increased significantly, compared with the results of other researchers (60–100 MPa) [15,42]. The flexural strength of 0.4 GNFs/0.1 CNTs composites with nano-carbon co-reinforced was also slightly higher than that of the composites with graphene or carbon nanotubes alone, which also reflected the synergistic enhancement of the three-dimensional structure composed of one-dimensional graphene and two-dimensional carbon nanotubes [25,26]. The compressive elastic modulus (10–14 GPa) of sintered composite was in the range of the elastic modulus of the human bone (dense bone 2–20 GPa [16]). It was revealed from Table 4 that the compressive strength and shear strength of 0.5-GNFs composite in all Nano-C reinforced titanium matrix/HA composites are slightly higher, which is related to the structure of graphene and carbon nanotubes. Because entanglement and agglomeration are more prone to occur in tubular CNTs if compared with lamellar GNFs, this in turn reduces the mechanical properties of the sintered composite.

### 3.4. Fracture Surface Analysis of the Sintered Nanocomposites

**Figure 11:**
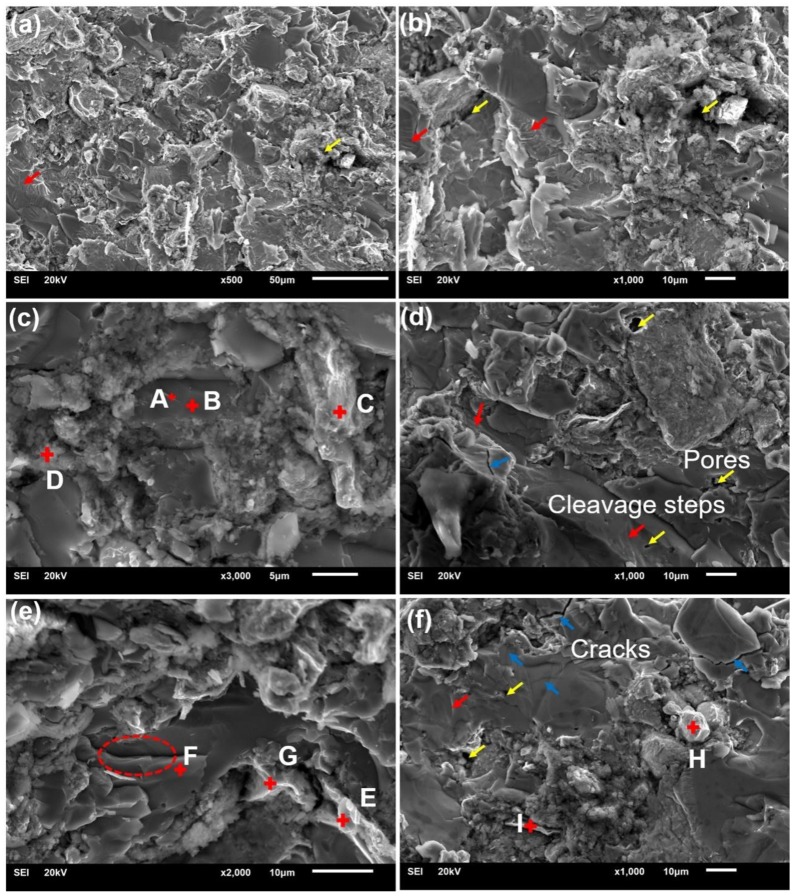
SEM micrographs of shear fracture surfaces of (**a**) the titanium alloy/HA nanocomposites; (**b**,**c**) the 0.5-CNTs nanocomposites; and (**d**,**e**) the 0.5-GNFs nanocomposites; (**f**) the 0.4 CNTs/0.1 GNFs nanocomposites respectively.

Figure 11 shows the micromorphology of shear fracture of nano-carbon reinforced titanium matrix/HA composite. It was seen that the fracture surface morphology of sintered composites is composed of lamellar materials, granular polymers, and agglomerated particles. As shown in Figure 11c,e, Table 5, the layered substances B and F mainly contained C, Ti, O, etc., and were titanium and oxides of titanium. Moreover, the layered substances may also contain a small amount of titanium carbide, but its presence is not detected in the XRD results (Figure 4) due to its low content. A separate white tube (Spot A) may be carbon nanotube, and its composition was similar to that of B. Because the surrounding layered material was also tested during testing spot A. In addition, some white lamellar substances (spot E, G, I) were present on the fracture surface, and mainly contained C, Ti, Nb, O, etc. The white lamellar substances were considered to graphene sheet layer, and some matrix substance were present on the surface of the graphene sheet layer. The C element was also detected elsewhere in the samples, but no significant presence of nano-carbon was observed, which was because that the nano-carbon is coated with other phases. Moreover, the existence of nano-carbon was found on the surface of the Ti or HA composites in the many literatures [20,21,22]. Due to incomplete sintering, there were still some large agglomerated particles in the composite, as indicated by spot H in Figure 11f. In addition, a large amount of granular particles (C, D) were present on the fracture surface, and mainly contained Ti, Nb, C, O, Ca, P, etc. 

On the other hand, the analysis found that shear fracture surface of composites has a cleavage step (indicated by the red arrows). The shear fracture belonged to the cleavage fracture, and researchers also found that there are some cleavage steps and river patterns on the compression fracture surface of Ti-5/10%HA composite [16]. Zhang et al. revealed that local cleavage fractures are mainly caused by weak bending between thin-walled walls [16]. At first, microscopic cracks appeared at the edges between the microscopic pores, and then expanded with the increase of pressure during the compression process. Therefore, macroscopic cracks occurred, and the composites broke when cracks reached a certain level [16,41]. In addition, studies have shown that the composite exhibits morphology of intergranular fracture when CaTiO_3_, Ca_3_(PO_4_)_2_ and Ti*_x_*P*_y_* are formed at the boundary of titanium particles [7]. There were mainly two kinds of pores on the surface of the sintered composites (indicated by yellow arrows): interconnected pores mainly distributed at the interface of composites and isolated pores on the lamellar material. The existence of interconnected pores was caused by the gas evolution from the reactions of HA and Ti, and the difference in thermal expansion coefficient between titanium alloys and HA. Isolated circular micropores on the lamellar material were caused by necking during sintering [29]. There were also some macroscopic cracks (indicated by blue arrows) on the fracture surface of composites, which were mainly distributed on the lamellar materials. It can be seen from Figure 11b,e that there are some dimples in the layered materials. This was caused by the inconsistency of the deformation between second phase particles and matrix under the stress during deformation process, which leaded to nucleation, growth, and fracture of microporous, and represented certain ductile fracture characteristics. It can be seen from the red circle in Figure 11e that the lamellar material is peeled off due to the crack tail stripping caused by deformation or agglomeration that occurs during sintering [15,43].

### 3.5. In Vitrobiocompatibilityassessment of the Sintered Nanocomposites

**Figure 12:**
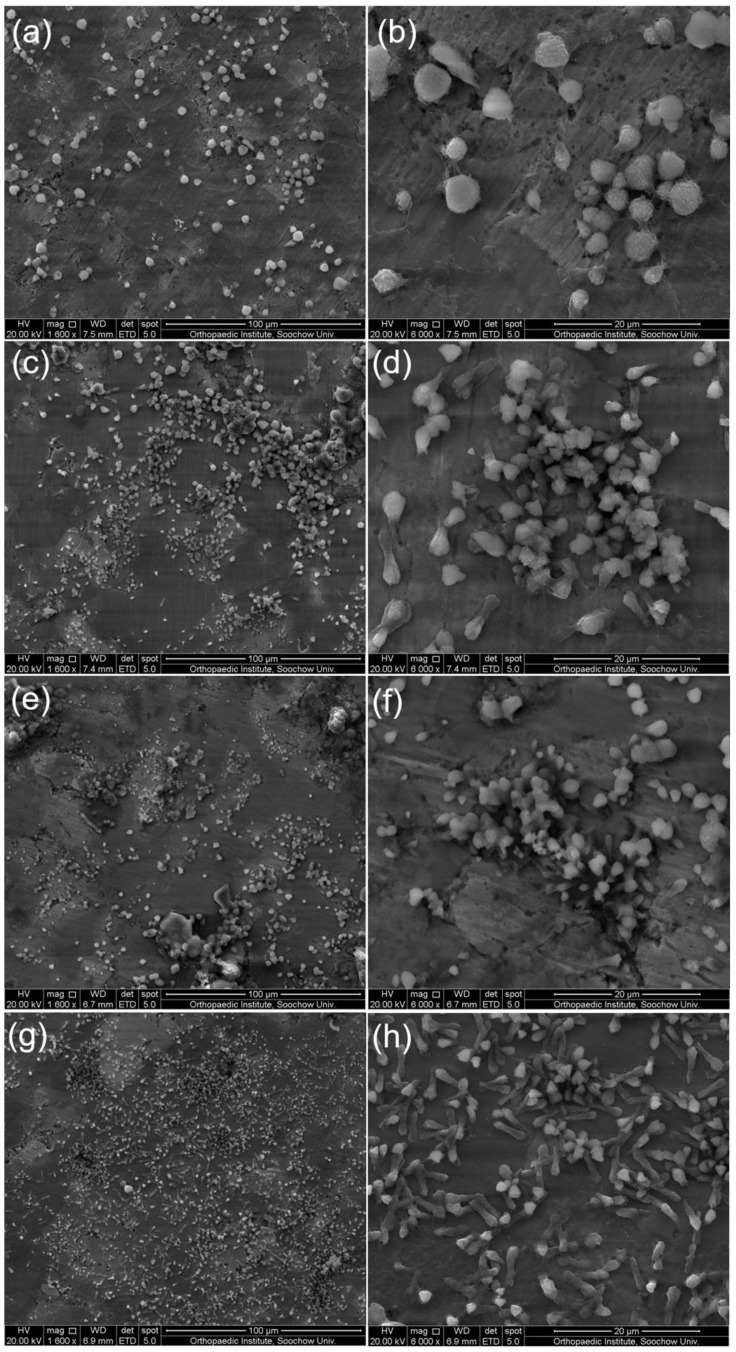
Morphology of osteoblast cells after three days culture on the surface of the sintered composites: (**a**,**b**) Ti-Ta-Nb-Zr/HA; (**c**,**d**) 0.5 CNTs; (**e**,**f**) 0.5 GNFs; (**g**,**h**) 0.4 CNTs/0.1 GNFs.

In vitro biocompatibility of nano-carbon reinforced titanium matrix/HA composites was evaluated by culturing MC3T3-E1 cells on the surface of composites. Figure 12 shows typical SEM images of MC3T3-E1 cells incubated on the surface of sintered composites after three days. It can be seen that MC3T3-E1 cells adhere well to the surface of sintered composites. Figure 11a,b shows that cells on the surface of titanium alloy/HA composite have a typical spherical morphology, which was the initial state of cell adhesion [34]. In contrast, cells on the surface of CNTs (Figure 11d) and GNFs (Figure 11f) reinforced titanium matrix/HA composites appeared approximate spindle-like structure, which showed better adhesion and proliferation of cells on the surface of composites [34]. The researchers also found that the composites added nano-carbon are ideal materials for scaffolds of bone tissue engineering, and beneficial to the proliferation and differentiation of human fetal osteoblastic cell line (hFOB 1.19) [23]. It was seen from Figure 11d that there are some pseudopods around the cells of spindle-shaped structure [34]. Moreover, Figure 11b,f,h showed that there are some aggregation of adherent cells, whose formation is related to the morphology and composition of composites. Because the surface morphology, surface energy, surface roughness and chemical composition of sintered composites can all affect the cell proliferation [44]. Kumar et al. found the existence of lamellipodia and filopodia when the Saos-2 osteoblasts were cultured on the surface of HA/Ti composites [33]. Moreover, filamentous pseudopodia can reflect the interaction of cells and material surfaces before migration and adhesion. The existence of lamellipodia indicated that cell migration and stretching are essential processes for cell proliferation [33]. In addition, it can be clearly seen from Figure 11g,h that the cells cultured on the surface of 0.4 GNFs/0.1 CNTs composite in the all sintered composites have more dense distribution and typical spindle-shaped morphology. This reflected its superior cell compatibility, and indicated that the synergistic effect of GNFs and CNTs can promote the proliferation of cells cultured on the surface of composites.

**Figure 13:**
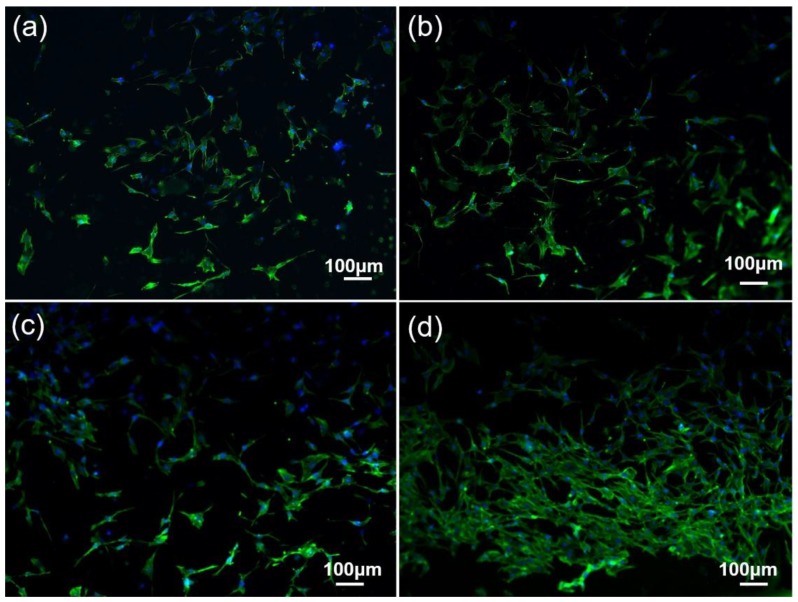
Representative fluorescent microscopic images of MC3T3-E1 cells grew for three days on the surface of sintered composites. (**a**) Ti-Ta-Nb-Zr/HA; (**b**) 0.5 CNTs; (**c**) 0.5 GNFs; (**d**) 0.4 CNTs/0.1 GNFs. The green and blue colors are showing the actin filament and nucleus, respectively.

Figure 12 shows fluorescence microscopy images of cells cultured on the surface of sintered composite after three days. Blue staining revealed the distribution of the cell nucleus on the surface of composites, and the green staining reflected the distribution of cytoplasm on the surface of the composites [45]. It can also be seen from Figure 12 that there are a large number of adherent cells on the surface of composites, and the distribution of adherent cells incubated on the surface of 0.4 GNFs/0.1 CNTs composite is denser. The green staining areas in Figure 13 were almost distributed on the entire surface of composites and connected to each other, which indicated that the sintered composites can promote cell adhesion and proliferation. On the one hand, the excellent biocompatibility of nano-carbon reinforced titanium matrix/HA composites was because of the presence of nano-hydroxyapatite and its products [33]. Nano-grade apatite particles can effectively combine with serum proteins and growth factors [34]. On the other hand, the presence of GNFs and CNTs can prompt the formation of mineralized necks of composites after transplantation, stimulate biomineralization, and enhance the biological activity of composites [18,34,35,46]. Moreover, researchers revealed that the presence of nano-carbon can adsorb various macromolecules such as proteins, which is beneficial to promote cell proliferation [18,34]. However, some studies have mentioned that graphene has an adverse effect on MG63 cells due to the general toxicity mechanism of carbon-based reactive oxygen species [35].

## 4. Discussion

Studies have shown that HA will decompose at about 800 °C in titanium matrix/HA composites, the reaction process is shown as Equations (1) and (2) [10,16]. Then, HA will continue to decompose or react with titanium, resulting in the occurrence of complex chemical reactions shown as Equations (3)–(7) as the temperature increases [7,10,17,29,38]. However, only Ti_17_P_10_, CaTiO_3_, Ca_3_(PO_4_)_2_, TiO_3_, ZrO_2_, TiO_3_, etc. were detected in the experiment. TiO_3_ and ZrO_2_ were formed by the reaction of titanium and zirconium with oxygen in the environment, respectively. Although the experiment was carried out in the vacuum environment, the oxygen in the sintering furnace was not completely removed. The researchers also found that titanium atoms tend to oxidize to form amorphous or crystalline TiO_2_ at the HA/Ti interface during high temperatures sintering [10,40,47]. The oxidation kinetics of titanium were determined by the adsorption rate of oxygen. At first, oxygen, considered as interstitial atom, diffused into the titanium lattice until reaching saturation levels. Then titanium atoms began to oxidize, but the diffusion rate of the oxygen was reduced when TiO_2_ was continuously formed [48]. Ye and Chu et al. also revealed that the presence of titanium atoms promotes the dehydroxylation of hydroxyapatite, and oxygen atoms diffuse into the titanium metal matrix to form titanium oxide in this case [49,50].

(1) $$Ca_{10}{\left(PO_{4}\right)}_{6}{\left(OH\right)}_{2}=Ca_{10}{\left(PO_{4}\right)}_{6}{\left(OH\right)}_{2-2x}O_{x}+xH_{2}O(g)\;$$

(2) $$Ca_{10}{\left(PO_{4}\right)}_{6}{\left(OH\right)}_{2}=Ca_{10-x}{\left(PO_{4}\right)}_{6}{\left(OH\right)}_{2-2x}+xCaO+xH_{2}O(g)\;$$

(3) $$Ca_{10}{\left(PO_{4}\right)}_{6}{\left(OH\right)}_{2-2x}O_{x}=2Ca_{3}(PO_{4})+Ca_{4}P_{2}O_{9}+(1-x)H_{2}O(g)\;$$

(4) $$Ca_{10}{\left(PO_{4}\right)}_{6}{\left(OH\right)}_{2}=2Ca_{3}(PO_{4})+Ca_{4}P_{2}O_{9}+H_{2}O(g)\;$$

(5) $$Ca_{10}{\left(PO_{4}\right)}_{6}{\left(OH\right)}_{2}+TiO_{2}=3Ca_{3}(PO_{4})+CaTiO_{3}+H_{2}O(g)\;$$

(6) $$6Zr+O_{2}(g)+2Ca_{3}(PO_{4})=6CaZrO_{3}+4P\;$$

(7) $$Ti+1/2O_{2}(g)+Ca_{3}(PO_{4})=3CaTiO_{3}+2P\;$$

It can be clearly seen from Table 6 that grain size of Ta/Nb and α-Ti after sintering is significantly increased, which is mainly due to the gradual growth of crystal grains at the middle and later stages of sintering process. Grain growth resulted from the movement of grain boundary, and its driving force was the free energy of grain boundary. Therefore, crystal surface areas decreased as crystal grains grew, which prompted densification of composites to some extent. It was revealed that the three strong peaks of α-Ti shift to the left after sintering and the three strong peaks of Ta/Nb shift to the right, which is because that other elements diffuse into their crystal lattice according to the Bragg formula. This also verified the results analysis of EPMA that Zr elements tended to diffuse into the crystal lattice of Nb and form solid solution. Moreover, studies have also shown that compared with that of pure-Ti, the strength of α-Ti in porous Ti/HA composites after vacuum hot pressing reduced, which indicates that the lattice of titanium is distorted. This suggested that the oxygen in the HA may diffuse into the lattice of titanium, change crystal structure of titanium, and play a role of solution strengthening [14]. However, the diffusion of oxygen resulted of the occurrence of brittle fracture at the sintered necks of titanium when the porosity was high [14].

In addition, the existence of Ca_5_(PO_4_)_3_(OH), Ca_4_O(PO_4_)_2_, CaTi_4_(PO_4_)_6_, etc., which were mentioned in other literatures [15,16,51,52,53], were not detected by XRD (Figure 4). Moreover, a small amount of Ti_17_P_10_, CaTiO_3_, Ca_3_(PO_4_)_2_ were detected. On the one hand, this was due to adjustment of the sintering method and reduction of HA content. In fact, SPS sintering is a pressure-assisted sintering method based on spark discharge sintering between particles. It is heated by the spark plasma discharge mechanism and heat transfer mechanism during sintering [16]. On the other hand, the addition of Ta, Nb, Zr, nano-carbon, etc. can also reduce reaction degree to a certain extent, which was because the addition of second phases can prevent direct contact of titanium with HA and reduce the contact areas. Other researchers have also mentioned that controlling the sintering process during SPS process can preserve the presence of HA and Ti, and weaken the complex chemical reactions between HA and Ti [33]. In the experiment, the GNFs pulling out and the bridging effect of CNTs promoted the improving of toughness of composites. The presence of broken graphene sheets and carbon nanotubes were found on the fracture surface of composites. In addition, crack tail stripping was also beneficial to the increase of fracture toughness of composites [41]. Therefore, based on the study of microstructure and mechanical properties above, it can be found that the strengthening and toughening mechanisms existing in nano-carbon reinforced titanium matrix/HA composites mainly include second phase strengthening, grain refinement strengthening, solution strengthening, graphene extraction, carbon nanotubes bridging, crack tail stripping, etc. There were second phases in the composites, such as nano-carbon and lanthanum. Studies have shown that the existence of lanthanum not only capture the oxygen of composites but also promote grain refinement, which improve the performance of composite [31]. Due to the agglomeration of nano-carbon, the effect of second phase strengthening [20] was not obvious. In addition, the ball milling process also promoted the improving of performance of composites due to grain refinement strengthening and uniform distribution of element. In addition, the various elements in the composites interdiffused during the sintering process, resulting in the formation of solid solution, such as Nb (Zr).

## 5. Conclusions

1. Nano-carbon reinforced titanium matrix/HA biocomposites were successfully prepared by spark plasma sintering. Results showed that there are some new phases detected in sintered composites, such as β-Ti, Ti_17_P_10_, CaTiO_3_, Ca_3_(PO_4_)_2_, TiO_3_, ZrO_2_, etc., but the reactions that may occur during the preparation process are suppressed to some extent. TEM analysis proved the existence of elemental diffusion and chemical reactions in sintered composites. The shear fracture belonged to cleavage fracture, and there were obvious cleavage steps, pores, and some cracks on the surface of the composites.
2. Compared with the traditional hot press sintering, the mechanical properties of the composites prepared by SPS, such as density (>98%), compressive strength (847–1134 MPa), shear strength (178–228 MPa) and bending strength (190–220.15 MPa), have been significantly improved. The compressive strength and shear strength of 0.5-GNFs composite in all Nano-C reinforced Titanium matrix/HA composites were slightly higher. Because entanglement and agglomeration is more prone to occur in tubular CNTs than lamellar GNFs.
3. The strengthening and toughening mechanisms existing in nano-carbon reinforced titanium matrix/HA composites mainly included second phase strengthening, grain refinement strengthening, solution strengthening, graphene extraction, carbon nanotubes bridging, crack tail stripping, etc. In vitro cells proliferation test showed that nano-carbon reinforced titanium matrix/HA composites have good biocompatibility. Moreover, the addition of nano-carbon in the titanium matrix/HA composites was beneficial to promote cell adhesion and proliferation.

## Figures and Tables

**Table 1:** The ingredients list of the experiment (wt %).

Sample	HA	La	CNT	GNFs	Nb	Ta	Zr	Ti
#1	5	0.3	0	0	27.65	11.55	4.07	51.43
#2	5	0.3	0.5	0	27.51	11.49	4.05	51.15
#3	5	0.3	0	0.5	27.51	11.49	4.05	51.15
#4	5	0.3	0.4	0.1	27.51	11.49	4.05	51.15

**Table 2:** The dispersive X-ray spectroscopy (EDS) results (at. %) in Figure 3.

Spot	C	O	P	Ca	Ti	Zr	Nb	La	Ta
1	4.47	29.86	0.65	0.89	64.01	0.04	0.07	-	0.01
2	58.71	31.79	2.75	4.06	1.83	0.20	0.35	0.05	0.09
3	18.77	47.61	2.62	3.73	26.67	0.19	0.31	0.02	0.07
4	17.22	40.89	2.83	3.36	17.32	-	18.38	-	-
5	26.41	27.36	1.72	1.42	1.38	-	0.23	0.10	41.38
6	67.26	19.54	2.05	3.31	6.51	-	1.34	-	-
7	59.18	22.36	3.04	4.86	8.32	0.38	1.44	-	0.41

**Table 3:** The EDS results (at. %) for regions A-Ein Figure 5.

Region	O	P	Ca	Ti	Zr	Nb	C	La	Ta
Point 1	26.24	3.74	26.20	7.34	-	14.03	22.44	-	-
Point 2	49.13	1.77	18.08	5.21	1.91	2.89	18.66	-	2.35
Point 3	20.40	0.02	0.27	55.45	0.08	0.17	22.19	0.93	0.48

**Table 4:** Mechanical properties of the nanocomposites sintered at 1000 °C.

Sample	Density	Microhardness/HV	Compressive Strength/MPa	Shear Strength/MPa	Bending Strength/MPa	Pressive Modulus/GPa
Ti alloy/HA	98.56%	518.6	1134.97	228.7	220.15	13.22
0.5-CNTs	98.83%	454	847.58	186.24	190.53	10.31
0.5-GNFs	99.04%	400.7	1046.05	192.55	191.06	12.47
0.4 CNTs: 0.1 GNFs	99.61%	413.7	922.55	178.1	196.72	11.41

**Table 5:** The EDS results (at. %) for regions A-I in Figure 11.

Region	C	P	Ca	Ti	O	Nb	Zr	Ta
**A**	24.27	0.42	0.85	74.47	-	-	-	-
**B**	25.61	1.28	0.66	72.45	-	-	-	-
**C**	44.89	2.00	1.83	4.34	24.18	22.74	-	-
**D**	20.14	3.67	2.42	38.81	31.78	3.18	-	-
**E**	14.89	-	0.53	50.04	31.94	1.64	0.95	-
**F**	11.18	-	0.37	66.39	21.51	-	0.55	-
**G**	37.37	-	4.10	5.15	26.23	26.75	-	-
**H**	68.20	-	0.54	2.48	11.98	-	-	16.80
**I**	48.31	-	2.95	2.28	33.77	12.70	-	-

**Table 6:** Grain size of some phases in 0.5-GNFs nanocomposite calculated by XRD Pattern Processing & Identification software.

Element	Treatment Condition	2θ	Crystal Face	Grain Size/Å
α-Ti	milled	40.155/38.590/35.087	(101)/(002)/(100)	253/258/235
	sintered	39.770/38.523/34.846		303/359/327
Ta/Nb	milled	38.461/69.593/55.649	(110)/(211)/(200)	254/164/199
	sintered	38.523/69.680/55.657		359/197/289
